# Copper induces transcription of *BcLCC2* laccase gene in phytopathogenic fungus, *Botrytis cinerea*

**DOI:** 10.1080/21501203.2020.1725677

**Published:** 2020-02-11

**Authors:** U. V. A. Buddhika, S. Savocchia, C. C. Steel

**Affiliations:** National Wine and Grape Industry Centre, School of Agricultural and Wine Sciences, Charles Sturt University, Wagga Wagga, Australia

**Keywords:** *Botrytis cinerea*, laccases, inducers, copper, gene expression, enzyme activity

## Abstract

Laccases are one of many groups of inducible enzymes produced by the filamentous fungus, *Botrytis cinerea* during colonisation of host plant tissues. While the processes involved in laccase induction are not fully understood, Cupric ions (e.g. CuSO_4_) and gallic acid (GA) have been reported as laccase inducers. This study investigates laccases activities and the expression of three laccase genes (*BcLCC1, BcLCC2, BcLCC3*) in three *B. cinerea* isolates grown in laccase-inducing medium (LIM) supplemented with CuSO_4_ and GA. Laccase activity in culture filtrates with CuSO_4_ increased after 48 h of growth in LIM at 24°C. The induction of *BcLCC2* transcription was greatest at a concentration of 0.6 mM CuSO_4_, concentrations greater than 0.6 mM inhibited fungal growth. In contrast, no laccase induction was observed in the presence of GA. Liquid chromatography-mass spectroscopy (NanoLC ESI MS/MS) analysis confirmed the presence of a 63.4 kDa protein, the *BcLCC2* isoform in the culture filtrate with 0.6 mM CuSO_4_. Analysis of mRNA transcripts further showed *BcLCC3* was also inducible and the expression of *BcLCC2* and *BcLCC3* was isolate-dependent. In conclusion, CuSO_4_ induces a 63.4 kDa laccase in *B. cinerea* by induced transcription of the *BcLCC2* gene.

## Introduction

*Botrytis cinerea* [anam.] *Botryotinia fuckeliana* [teleom.], Pers.: Fr- (grey mould) is a devastating fungal pathogen that is responsible for grey mould in many fruits, vegetables and horticultural crops, particularly in temperate regions (Dean et al. 2012). *B. cinerea* attacks many plant organs, including leaves, stems and fruits, resulting in severe losses even after the crop has been harvested (Lorenzini et al. 2013). At least 1400 plant species are reported to be affected by *B. cinerea* (Elad et al. 2016). The infection process typically involves extracellular enzymes and metabolites of the pathogen (Nakajima and Akutsu 2014), including cell wall-degrading enzymes, non-specific phytotoxic metabolites (botrydial and botcinolides), boosting compounds of reactive oxygen species (ROS) and the plants’ hypersensitive response (Williamson et al. 2007; Shah et al. 2009; Amselem et al. 2011). The extracellular enzymes include β-glucosidase, pectin methylesterases, polygalacturonases, aspartate proteinase, benzyl alcohol oxidase and laccases (Rha et al. 2001; Valette-Collet et al. 2003; Nakajima and Akutsu 2014).

Laccases (benzenediol: oxygen oxidoreductase, EC 1.10.3.2) are found in vascular plants, fungi, bacteria and insects (Thurston 1994; Castilho et al. 2009), although the lignin-degrading white rot basidiomycetes are the main laccase producers (Rayner and Boddy 1988). Laccases belong to the largest subgroup of blue multi-copper oxidases (MCOs) and typically show phenoloxidase activity. During the oxidation process, laccases use oxygen as an electron acceptor to remove hydrogen radicals from a phenolic hydroxyl group and other similar molecules (Thurston 1994; Solomon et al. 1996). In addition, laccases oxidise a wide range of substituted aromatic amines, N-heterocycles, phenothiazines and thiol groups. Laccases contain two to four copper atoms per enzyme molecule (subunit), contributing to their oxidation properties (Thurston 1994; Giardina et al. 2010). Copper atoms are located in three types of copper centres that can be distinguished by their spectroscopic and paramagnetic properties (Thurston 1994). Cupric ions (Cu^2+^) are crucial for forming metal-active sites in laccases; hence, laccases are classified as copper-containing oxidases.

Fungal laccases are either inducible or constitutively expressed (Litvintseva and Henson 2002). Laccase production in lignin-degrading fungi and some ascomycete fungi have been investigated with phenolic inducers, different carbon sources and metal ions (Palmieri et al. 2000; Manavalan et al. 2013; Kim et al. 2014). Cu^2+^ is reportedly a strong metal-inducers for both laccase transcription and activity (Collins and Dobson 1997) and are vital in forming metal-active sites in copper-containing enzymes (Passarini et al. 2015). The interaction between Cu^2+^ and the fungal mycelium in culture medium has been shown to cause a substantial increase in total laccase activity in wood-rotting fungi, basidiomycetes (Guillén and Machuca 2008; Makela et al. 2013; Kuhar and Papinutti 2014) and the ascomycetes fungus, *Aspergillus flavus* (Gomaa and Momtaz 2015).

Laccase induction has been reported in *B. cinerea* in response to phenolic compounds, particularly gallic acid (GA). However, an increase in laccase activity in *B. cinerea* was reported when copper was added to the culture medium (Slomczynski et al. 1995; Sbaghi et al. 1996). Different isoforms of the enzymes, including a 36 kDa protein with 70% sugar and a 38 kDa protein with 80% sugar have been reported to be induced in liquid cultures of *B. cinerea* in the presence of GA and grape juice, respectively (Marbach et al. 1984). In addition, a 75 kDa protein has been purified from the liquid cultures of *B. cinerea* in the presence of Cu^2+^ (Slomczynski et al. 1995), respectively. Copper induction of *B. cinerea* laccases and the involvement of copper inducible laccase in disease development has not been fully elucidated, although the involvement of GA-inducible laccases in disease development has previously been reported (Gigi et al. 1980, 1981). However, the occurrence of 13 putative laccase encoding genes has been reported (Van Kan et al. 2017), although the biological basis of the induction of these genes, especially the enzyme-gene linkage and type of inducers remain unknown. Therefore, this study hypothesised that GA and Cu^2+^ are responsible for inducing a particular laccase gene, thereby increasing laccase activity. The current study investigates laccase activities, and the expression of laccase genes using RT-qPCR and RT-PCR methods in *B. cinerea* grown artificial media treated with Cu^2+^ and GA. To investigate this, three laccase genes (*BcLCC1, BcLCC2, BcLCC3*) were considered based on the previously published work of Schouten et al. (2002), which describes the potential involvement of *BcLCC2* and *BcLCC3* in disease development.

## Materials and methods

### Isolates

Three *B. cinerea* isolates (TN080, VRU0005, and TN077), representative of high, low and moderate virulence, respectively (data not shown), were used in the study. The isolates were taken from the culture collection stored at the National Wine and Grape Industry Centre, Charles Sturt University, Australia. The *BcLCC2* gene from the three isolates was sequenced and the resulting sequences compared with NCBI GenBank sequences using BLAST. Sequences were deposited in GenBank with following accession numbers (TN080: MH789981, VRU0005: MH789982, and TN077: MH789980).

### Culture conditions and preparation of laccase-inducing medium (LIM)

The basal media for liquid cultures were prepared by following the protocol described by Slomczynski et al. (1995). LIM was prepared by supplementing the basal medium with CuSO_4_ and GA at concentrations of 0.35 mM and 0.5 mM, respectively.

### Induction of laccases by CuSO_4_ and GA

The three *B. cinerea* isolates were grown on potato dextrose agar (PDA) at 24°C. Sterile distilled water was added to the surface of 12-day-old sporulating cultures and spore suspensions were adjusted to 1 × 10^5^ spores/ml using a haemocytometer. A 100 µl spore suspension (1 x 10^5^ spores/ml) was inoculated into 30 ml of potato dextrose broth (PDB) in 100 ml conical flasks followed by incubation in a rotary shaker at 90 rpm at 24°C in the dark conditions. After 5 days, the mycelia were harvested using a sterilised Buchner funnel lined with sterilised filter paper. The mycelia were washed with sterile distilled water and transferred to LIM containing a mixture of CuSO_4_ and GA. Basal medium was used as a control. The cultures were incubated for 2 days at 24°C with agitation (240 rpm) in the dark. Aliquots of 2 ml were collected from the liquid cultures of inoculated LIM and the control flask within 12-h interval for up to 48 h and stored at −20°C for the determination of laccase activity and total protein. The experiment was performed in triplicates.

### Expression analysis of laccase genes in the presence of CuSO_4_ and GA

Transcripts of laccase genes were quantified for the three isolates 48-h post-inoculation (hpi) in LIM. The LIM were treated separately with CuSO_4_ and GA. The experimental units consisted of PDB as the control, the basal medium as the control (NoI), LIM + CuSO_4_, LIM + GA, and LIM + CuSO_4_ + GA. LIM and control media were inoculated with three isolates separately as described in the aforementioned section. The experiment was performed in three independent replicates. Mycelia from all liquid cultures were collected 48-h post-inoculation (hpi) to analyse the expression profiling of laccase genes (*BcLCC1, BcLCC2, BcLCC3*). Culture filtrates were also collected at the same time to determine laccase activity. Mycelia collected from PDB were excluded from gene expression analysis as no laccase activity was detected in PDB.

### Determination of the optimum copper concentration for the induction of laccase

*B. cinerea* isolate, TN077, in which the laccase genes were induced by both inducers was selected to determine the optimum concentration of CuSO_4_ for maximum laccase production. LIM supplemented with different concentrations of CuSO_4_ (0, 0.2, 0.4, 0.6, 0.8 mM) and inoculated with clean mycelia were prepared. The experiment was performed in triplicates. After 48-h incubation, mycelia were collected from each liquid culture using a Buchner funnel to determine fungal biomass and to investigate gene expression. Liquid filtrates were also collected from the same cultures at the same time to determine laccase activity and to perform polyacrylamide gel electrophoresis. The laccase protein was then purified from the liquid culture filtrate treated with 0.6 mM CuSO_4_ and followed by NanoLC ESI MS/MS analysis.

### Enzyme assays

Measurement of enzyme activity was based on the oxidative dimerisation of 2,2′-azino-bis (3-ethylbenzothiazoline-6-sulfonic acid) (ABTS) (Sigma). The reaction mixture contained 100 µl of ABTS (26 mg/ml), 800 µl of 0.1 M phosphate buffer (pH 6.0) and 100 µl of enzyme extract (culture filtrate) in a final volume of 1 ml. The reaction was incubated at room temperature (24°C) for 5 min, where the reaction was linear, and monitored by measuring absorbance at 436 nm (Guetsky et al. 2005) using a UV-visible spectrophotometer (Thermo Scientific Helios). One unit (U) of enzyme activity was defined as the amount of enzyme that converts one micromole of substrate per minute. Enzyme activities of all the samples were expressed as U ml^−1^. Total protein in culture filtrates was determined by the Bradford method (Bradford 1976).

### RNA extraction, cDNA synthesis and RT-qPCR

RNA was extracted from the fungal mycelia using the ISOLATE II RNA Plant Kit (Bioline) following the manufacturer’s guidelines. RNase-free DNase I was used to digest traces of contaminating genomic DNA. RNA quality and quantity were determined using a NanoDrop spectrophotometer (Thermo Fisher Scientific, Wilmington, DE, USA). cDNAs were synthesised from 1 µg of RNA using the High-Capacity cDNA Reverse Transcriptase Kit (Thermo Fisher Scientific, USA).

Laccase gene-specific primers were designed to measure the expression of laccase genes (supplementary Table). Aliquots of 2 µl from all cDNA samples were used for RT-qPCR in a 20 µl reaction mixture that contained 10 µl of 2x iTAQ Universal SYBR Green Supermix master mix (Bio-Rad), 0.5 µl of forward and reverse primers (Supplementary Table), and 7 µl of water. Two technical replicates were performed from each experimental unit. RT-qPCR was performed on a CFX96 real-time system (Bio-Rad) with cycling conditions of 3 min, initial denaturation at 95^°^C, 46 cycles for 10 s denaturation at 95^°^C, and 30 s annealing at 58°C, followed by melting curve analysis to confirm the specificity of PCR amplification. The relative mRNA expression levels (fold changes) of all laccases (*BcLCC1, BcLCC2*, and *BcLCC3*) were calculated using the ∆∆C_t_ method (Livak and Schmittgen 2001) and normalised to the expression of the ubiquitous housekeeping gene, ActinA (Simon et al. 2013; Kelloniemi et al. 2015). The mean gene expression in each treatment was normalised to the control (NoI).

### Determination of fungal biomass

Fungal biomass of the mycelia was determined by measuring the constant dry weight. Fungal mycelia were harvested onto filter paper (45 mm diameter Whatman No 2) under vacuum and kept at 75^°^C in an oven until a constant weight was achieved.

### Polyacrylamide gel electrophoresis

For purification of extracellular laccase, the collected liquid filtrates from the cultures of *B. cinerea* grown in different copper concentrations were thawed and centrifuged to remove precipitated polysaccharides. Each filtrate was concentrated using 30 kDa membrane filters (Merck Millipore, Amicon Ultra – 15 Centrifugal Filters, Castle Hill, NSW, Australia). Twenty microlitres of concentrated filtrate was mixed with 30 µl of NuPAGE LDS (4X) sample buffer (Novex, Life Technologies, USA) (pH 6.8), denatured at 95°C and 40 µl was loaded into each lane of a 12% precast gel (Criterion^TM^ TGX^TM^, BIO-RAD), followed by electrophoresis at room temperature using Tris-glycine buffer (pH 8.3) at 300 V (Laemmli 1970). The polyacrylamide gels were stained with Coomassie Brilliant Blue R-250 (Castle Hill, NSW, Australia) and visualised using a Gel Doc system (Bio-Rad). A high range molecular marker (Bio-Rad) was used for the determination of 75 kDa protein.

### Laccase purification from the fungal culture filtrate and identification

The liquid culture filtrate of TN077 collected from LIM supplemented with 0.6 mM CuSO_4_ was treated as described above in polyacrylamide gel electrophoresis analysis to remove precipitated polysaccharides followed by mixing with ammonium acetate in cold methanol. They were mixed in a ratio of one volume of the filtrate to five volumes of 0.1 M w/v ammonium acetate in cold methanol, kept at −20^°^C overnight (Vincent et al. (2006), and then centrifuged at 4000 rpm, for 20 min. The pellet was collected and washed with 5 ml cold acetone and dissolved in 30 µl of NuPAGE LDS (4X) sample buffer (Novex, Life Technologies, USA), followed by SDS/PAGE. The bands corresponding to 75 kDa were excised from the polyacrylamide gel and sent to the Australian proteome analysis facility (APAF; Macquarie University, Sydney) for identification using NanoLC ESI MS/MS. Detailed methods for NanoLC ESI MC/MC data acquisition are available in supplementary method.

### Statistical analysis

Normality of the distribution of data from enzyme assays, transcriptomics analysis and fungal biomass, and the constancy of residuals were confirmed using the normality test. The data of laccase activity and relative expressions of each laccase gene in each isolate in the presence and absence of different inducers were subjected to one-way analysis of variance (ANOVA), and the means of each treatment, with inducers were compared with the control, without inducers (NoI) using Dunnett’s mean comparison test. Laccase activity data and the relative expression of each laccase gene in TN077 and in the presence of each copper concentration were subjected to one-way analysis of variance (ANOVA), and the means in each concentration were compared using Tukey’s mean comparison test. Statistical significance was attained at the 5% probability level and all statistical analyses were performed using MiniTab 15.0 (MiniTab, Inc., State College, PA).

## Results

### Laccase activity in B. cinerea increases 48 hpi in LIM

**Figure 1.**
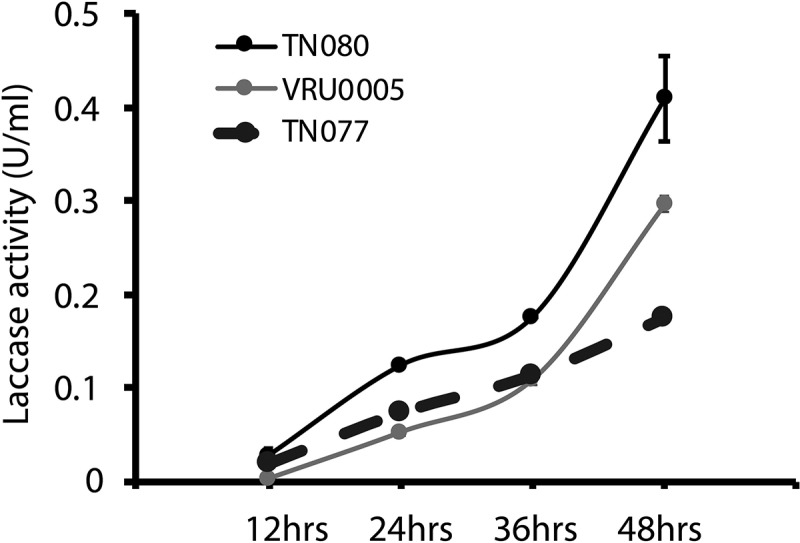
Effect of inducers on the laccase activity in three isolates of *Botrytis cinerea* (TN080, VRU0005, TN077) in laccase-inducing medium; kinetics of laccase activity was measured within 12-h incubation of *Botrytis cinerea* isolates in media supplemented with combination of inducers (GA and CuSO_4_)

Laccase activity in the culture filtrates of all isolates in *B. cinerea* increased in response to a mixture of CuSO_4_ and GA in LIM 48 hpi (Figure 1).

### Copper induces laccase activity and BcLCC2 and BcLCC3 gene expressions

**Figure 2.**
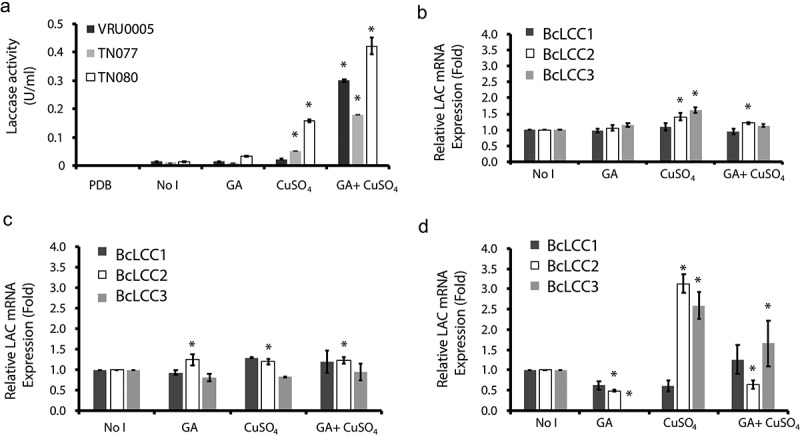
Effect of inducers (CuSO_4_ and GA) apart on the laccase activity (a) and the normalised expression levels of *BcLCC1, BcLCC2, BcLCC3* of *Botrytis cinerea* isolates (TN080), (b); VRU0005, (c); TN077, (d). The laccase-inducing medium was supplemented with the inducers, GA and CuSO_4_, and the control (NoI) was composed of basal medium without inducers. Error bars represent the standard error calculated from three biological replicates. Columns represent the three isolates in each inducer and columns with (*) are significantly different from the control of each isolate at p < 0.05 (Dunnett’s mean comparison test)

In the presence of both inducers separately, copper induced the expression of laccase genes and increased the enzyme activity in *B. cinerea*. All isolates, except VRU0005, showed significantly (p < 0.05) increased laccase activity with CuSO_4_, whereas no significant increases in laccase activity in the presence of GA occurred for any of the isolates (Figure 2(a)). As a response to CuSO_4_, *B. cinerea* isolates showed different amounts of laccase activities, with TN080 the greatest (p < 0.05) (Figure 2(a)), and showed a significant (p < 0.05) increase in laccase activity when the medium contained a mixture of CuSO_4_ and GA (Figure 2(a)).

In terms of relative gene expression, all isolates showed significantly different expressions of *BcLCC2* and *BcLCC3* genes in the presence of CuSO_4_ with the greatest (p < 0.05) in TN080 (Figure 2(b)). Conversely, no expression of laccase genes was detected as a response to GA. However, in VRU0005, expression of only the *BcLCC2* was evident (p < 0.05) with both inducers, whereas there was no significant difference in the expression of *BcLCC1* and *BcLCC1*3 (Figure 2(c)). However, in TN077, the expression of both *BcLCC2* and *BcLCC3* was significantly different (p < 0.001) in the presence of CuSO_4_, whereas a significant (p < 0.015) down-regulation was observed in *BcLCC2* and *BcLCC3* in the presence of GA (Figure 2(d)). For all isolates, the expression of *BcLCC2* was induced by CuSO_4._ However, the expression of *BcLCC1* was constant as observed in all isolates.

### Copper increases laccase activity and induces BcLCC2 transcription in TN077 at an optimum concentration of 0.6 mM

**Figure 3.**
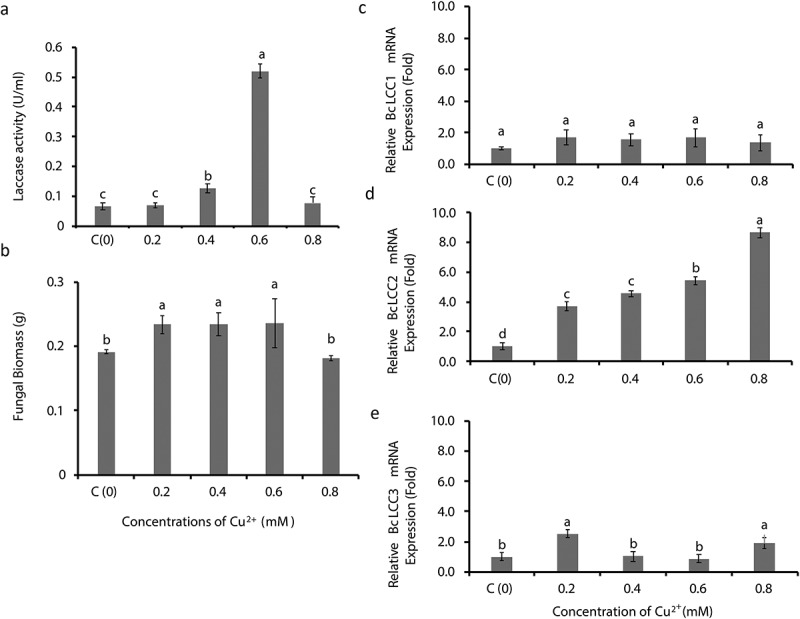
Copper-inducible laccase production in *Botrytis cinerea* (TN077) in the medium supplemented with different concentrations of CuSO_4_; (a) Effects of different concentrations of copper on laccase activity, (b) fungal biomass, and expression levels of laccase genes, (c) *BcLCC1*, D) *BcLCC2* and E) *BcLCC3* in *Botrytis cinerea* (TN077). The control medium was composed of basal medium with no inducers (NoI). Error bars represent the standard error calculated from three independent replicates and different letters (a,b,c,d) represent significant differences in mean value of each column at p < 0.05 (Tukey’s mean comparison test, n = 3)

Laccase activities in TN077 gradually increased with increasing concentration of copper, with the greatest (p < 0.001) at 0.6 Mm (Figure 3(a)). However, laccase activities were not significantly different between the highest, 0.8 mM and low concentrations (0 and 0.2 mM) (Figure 3(a)).

Fungal biomass was significantly increased (p < 0.05) when the culture medium was supplemented with 0.2 mM CuSO_4,_ and this remained the same with increasing concentrations up to 0.6 mM (Figure 3(b)). However, in the concentration between 0.8 mM and 0 mM (control), the fungal biomass was not significantly different (p > 0.05) and follows the same pattern of laccase activities as shown in the same concentration (Figure 3(a)).

Significant expression of *BcLCC2* (p < 0.001) (Figure 3(d)) and *BcLCC3* (p < 0.001) (Figure 3(e)) was observed along with different concentrations of CuSO_4_, whereas there was no significant expression of *BcLCC1* (p > 0.05 (Figure 3(c)). However, *BcLCC2* expression was concentration-dependent with a five-fold increase at 0.6 mM CuSO_4_, and *BcLCC2* showed a significant (p < 0.001) up-regulation relative to *BcLCC3*. The greatest laccase activity with a 40-fold increase of *BcLCC2* expression and fungal biomass was observed at 1 mM concentration (data not shown).

### NanoLC ESI MS/MS confirms BcLCC2 as copper inducible laccase with the molecular mass 63.4 kDa

**Figure 4.**
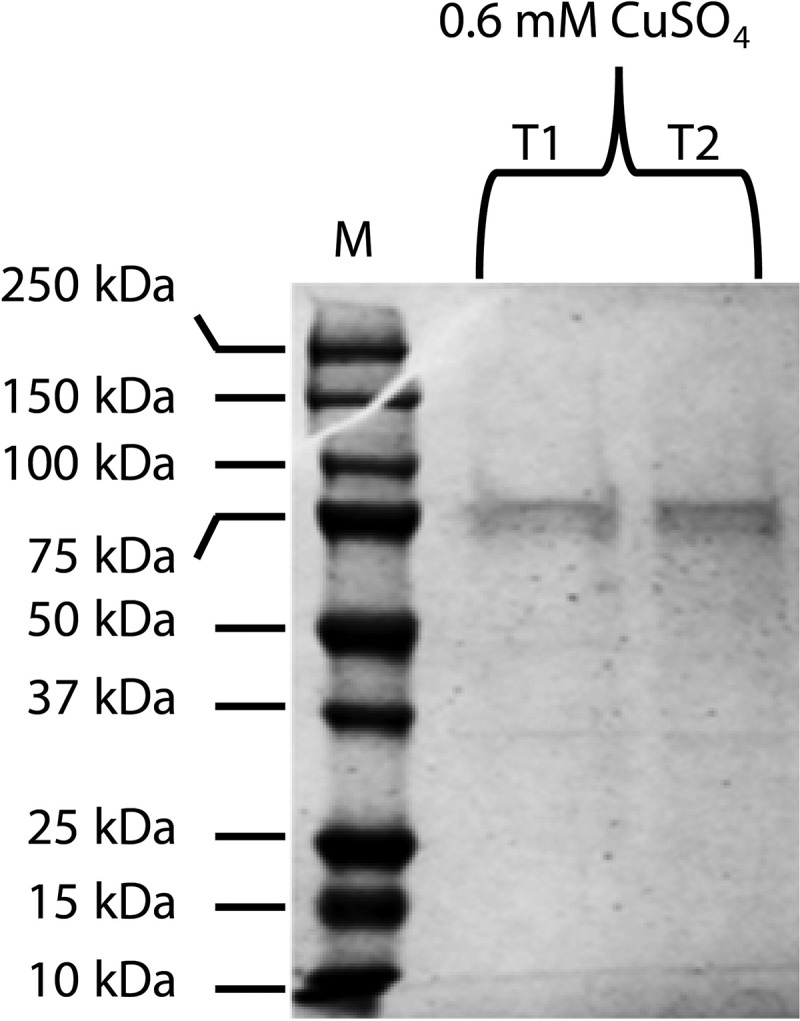
SDS-PAGE analysis for the purified protein from the two separate liquid culture filtrates (T1, T2) of *Botrytis cinerea* supplemented with 0.6 mM CuSO_4_. The basal medium with 0 mM CuSO_4_ and PDB as control media and M = protein molecular weight marker

SDS/PAGE resulted in a single protein band of ≈75 kDa in that supplemented with 0.6 mM CuSO_4_ (Figure 4). ESI/MS/MS confirmed the molecular mass of this band as 63.4 kDa, and the peptides matched with LAC2-BOTFU Laccase-2 OS = *Botryotinia fuckeliana* GN = LAC2, and the score sequence coverage was 994 (individual ions scores > 37 indicate identity or extensive homology (p < 0.0059)).

## Discussion

The lack of knowledge of the biological basis of the induction and expression of laccase genes in *B. cinerea* limits our understanding of their role in grey mould development. This study examined the expression of laccase genes (*BcLCC1, BcLCC2* and *BcLCC3*) and enzyme activity in *B. cinerea* with respect to inducers, GA and CuSO_4_. Laccase activities of *B. cinerea* isolates peaked after 48-h incubation in LIM, when the culture medium was supplemented with a mixture of CuSO_4_ and GA. The expression of laccases genes when tested in the presence of each inducer alone, CuSO_4_ rather than GA induced laccase activity and the expression patterns of *BcLCC2* depended on the isolate. The synthesis and secretion of laccases in response to Cu^2+^ have been reported to be species and isolate dependent (Piscitelli et al. 2011; Park et al. (2015). The concentration of Cu^2+^ required for laccase induction varied between 0.01 and 1 mM, and that this depended on the fungal species (Passarini et al. 2015) as demonstrated in white rot fungi (Palmieri et al. 2000; Fonseca et al. 2010) and ascomycetes such as *Aspergillus flavus* (Gomaa and Momtaz 2015) and *G. graminis* (Litvintseva and Henson 2002). The induction of laccase in *Dichomitus squalens* has an optimum Cu^2+^concentration as low as 0.06 mM, whereas the marine filamentous non-heterocystous cyanobacterium, *Phormidium valderianum*, is negatively affected when the concentration of Cu^2+^ is greater than 0.01 mM (Kannaiyan et al. 2012). However, this is not always the case. The growth of some isolates of wood rotting fungi is stimulated with low concentrations of Cu^2+^, while others may be inhibited by even the lowest concentration (Guillén and Machuca 2008). From our study, it appears that a copper concentration of 0.6 mM is the optimum concentration for laccase induction in *B. cinerea*.

*B. cinerea* stimulated fungal growth in a dose depended manner up to 0.6 mM Cu^2+^. However, concentrations beyond this become inhibitory to fungal growth as a result of oxidative stress. This can be explained as Cu^2+^ mediated oxidative stress, which is extremely toxic to fungal cells (Fernández-Larrea and Stahl 1996; Manzl et al. 2004; Kannaiyan et al. 2012). As Cu^2+^stimulates fungal growth at concentrations below 0.6 mM, it is reasonable to suggest that there is a correlation between laccase activity and fungal biomass, unless the fungal growth is affected by the copper toxicity. Although 1 mM is toxic to fungal biomass, the dramatic increase of laccase activity and the induction of *BcLCC2* at this concentration could imply the over-expression of laccase to alleviate copper toxicity. It has been reported that fungi produce laccases to scavenge ROS to protect themselves from oxidative stresses caused by free Cu^2+^ (Stohs and Bagchi 1995) and, as a defence mechanism against copper (Viswanath et al. 2008). This scenario suggests that *BcLCC2* might be the stress-regulated laccase in *B. cinerea* as previously suggested by Schouten et al. (2002), although further investigation into this is required.

The consistent expression of *BcLCC2* along with different concentrations of Cu^2+^ indicates that *BcLCC2* is the copper-responsive gene. Laccase 2 has been reported to be the copper-inducible gene in *Gaeumannomyces graminis* (Litvintseva and Henson 2002), whereas *Pleurotus ostreatus* has more than one copper-responsive gene (Palmieri et al. 2000). *BcLCC3* was also found to be inducible, although the isozyme was not found. *BcLCC2* isozyme was identified from the liquid culture filtrate treated with 0.6 mM copper. The molecular mass of *BcLCC2* was confirmed to be 63 kDa in size, although 75 kDa laccase protein has been reported before (Slomczynski et al. 1995). The present study, however, demonstrated the molecular mass75 kDa before ESI/MS/MS analysis, which appears to concur with the findings of Slomczynski et al. (1995). This difference of the molecular mass could be due to the nature of the glycoprotein (Morozova et al. 2007; Maestre-Reyna et al. 2015), the function is presumably to confer thermostability of laccases up to 70°C (Yaropolov et al. 1994).

Most fungal laccases are inducible, although at least one laccase isozyme can be produced constitutively (Baldrian 2006). We observed the expression of *BcLCC1* to be constitutive, which explains why minor laccase activity was observed with no inducers. Besides the type of inducers, *in-vitro* fungal laccase production is dependent on culture conditions, nutrient levels, medium composition, fungal isolates or the strain, and developmental stage and the time of incubation of the fungus (Viswanath et al. 2014; Garrido-Bazán et al. 2016). Despite the fact that Cu^2+^ induced increased laccase activity, CuSO_4_ in conjunction with GA resulted in the greatest laccase activity. Similarly, increased laccase activities in *Ganoderma lucidum* (Manavalan et al. 2013), *Trametes* spp. (Jang et al. 2006) and *Pycnoporus sanguineus* (Ramirez-Cavazos et al. 2014) have been reported in the presence of combinations of different inducers, although the reason for this is not yet known.

The gene induction and the expression of laccase protein could be attributed to internal regulation mechanisms such as micro RNAs (miRNAs). Copper-miRNA is a systemic regulator of copper enzymes as microRNA expression patterns have shown negatively correlate with the accumulation of copper protein transcripts as reported in *Arabidopsis* (Abdel-Ghany and Pilon 2008; Delye 2013). Jin and Wu (2015) observed in *B. cinerea* that up-regulated miRNAs inhibited the expression of their target genes involving the post-transcriptional modifications. In *B. cinerea*, copper-miRNA might have been involved in the regulation of the transcription of *BcLCC2* in the presence of copper. However, this concept is yet to be investigated.

In conclusion, this study confirms that *BcLCC2* and *BcLCC3* in *B. cinerea* are induced in the presence of Cu^2+^. *BcLCC2* induction is a maximum at a Cu^2+^ concentration of 0.6 mM, and at higher concentrations, fungal growth is inhibited. However, the expression of the target gene apparent to be induced by a Cu^2+^ dependent internally regulated mechanism resulting in different laccase activities and expression patterns depending on the isolate. Copper induction of *BcLCC2* transcripts encodes a 63.4 kDa isoform of the enzyme in *B. cinerea* at the early stage of growth in a liquid culture. This study provides novel insight into *BcLCC2* induction in which Cu^2+^ as the inducer for the expression of *BcLCC2*-63.4 kDa enzyme, hence establishes an enzyme-gene linkage relevant to the type of inducer. However, such a link for *BcLCC3* and its induction as a response to a relevant inducer remains to be elucidated in future research and discovery of specific inducers for individual laccases may provide a better platform for understanding the biological basis of laccase expression during different stages of the disease development.

## Supplementary Material

Supplemental Material

## Data Availability

The data described in this article are openly available in the Open Science Framework at DOI:10.17605/OSF.IO/TPA6U.

## Supplementary material

The supplementary data for this article can be accessed here.

## References

[cit0001] Abdel-Ghany SE, Pilon M. 2008. MicroRNA-mediated systemic down-regulation of copper protein expression in response to low copper availability in Arabidopsis. J Biol Chem. 283:15932–15945. 18408011 10.1074/jbc.M801406200 PMC3259626

[cit0002] Amselem J, Cuomo CA, van Kan JAL, Viaud M, Benito EP, Couloux A, Coutinho PM, de Vries RP, Dyer PS, Fillinger S, et al. 2011. Genomic analysis of the necrotrophic fungal pathogens *Sclerotinia sclerotiorum* and *Botrytis cinerea*. PLoS Genet. 7:e1002230. 21876677 10.1371/journal.pgen.1002230 PMC3158057

[cit0003] Baldrian P. 2006. Fungal laccases - occurrence and properties. Fems Microbiol Rev. 30:215–242. 16472305 10.1111/j.1574-4976.2005.00010.x

[cit0004] Bradford MM. 1976. A rapid and sensitive method for the quantitation of microgram quantities of protein utilizing the principle of protein-dye binding. Anal Biochem. 72:248–254. 942051 10.1016/0003-2697(76)90527-3

[cit0005] Castilho FJ, Torres RA, Barbosa AM, Dekker RF, Garcia JE. 2009. On the diversity of the laccase gene: a phylogenetic perspective from *Botryosphaeria rhodina* (Ascomycota: fungi) and other related taxa. Biochem Genet. 47:80–91. 19160039 10.1007/s10528-008-9208-0

[cit0006] Collins PJ, Dobson A. 1997. Regulation of laccase gene transcription in *Trametes versicolor*. Appl Environ Microbiol. 63:3444–3450. 16535685 10.1128/aem.63.9.3444-3450.1997 PMC1389241

[cit0007] Dean R, Van Kan JA, Pretorius ZA, Hammond-Kosack KE, Di Pietro A, Spanu PD, Rudd JJ, Dickman M, Kahmann R, Ellis J, et al. 2012. The top 10 fungal pathogens in molecular plant pathology. Mol Plant Pathol. 13:414–430. 22471698 10.1111/j.1364-3703.2011.00783.x PMC6638784

[cit0008] Delye C. 2013. Unravelling the genetic bases of non-target-site-based resistance (NTSR) to herbicides: a major challenge for weed science in the forthcoming decade. Pest Manag Sci. 69:176–187. 22614948 10.1002/ps.3318

[cit0009] Elad Y, Pertot I, Prado AMC, Stewart A. 2016. Plant hosts of *Botrytis* spp. In: Fillinger S, Elad Y, editors. Botrytis – the Fungus, the pathogen and its management in agricultural systems. Cham: Springer International Publishing; p. 413–486.

[cit0010] Fernández-Larrea J, Stahl U. 1996. Isolation and characterization of a laccase gene from *Podospora anserina*. Mol Gen Genet MGG. 252:539–551. 8914515 10.1007/BF02172400

[cit0011] Fonseca MI, Shimizu E, Zapata PD, Villalba LL. 2010. Copper inducing effect on laccase production of white rot fungi native from Misiones (Argentina). Enzyme Microb Technol. 46:534–539. 25919631 10.1016/j.enzmictec.2009.12.017

[cit0012] Garrido-Bazán V, Téllez-Téllez M, Herrera-Estrella A, Díaz-Godínez G, Nava-Galicia S, Villalobos-López MÁ, Arroyo-Becerr A, Bibbins-Martínez M. 2016. Effect of textile dyes on activity and differential regulation of laccase genes from *Pleurotus ostreatus* grown in submerged fermentation. AMB Express. 6:93. 27718214 10.1186/s13568-016-0263-3 PMC5055507

[cit0013] Giardina P, Faraco V, Pezzella C, Piscitelli A, Vanhulle S, Sannia G. 2010. Laccases: a never-ending story. Cell Mol Life Sci. 67:369–385. 19844659 10.1007/s00018-009-0169-1 PMC11115910

[cit0014] Gigi O, Marbach I, Mayer AM. 1980. Induction of laccase formation in *Botrytis*. Phytochemistry. 19:2273–2275.

[cit0015] Gigi O, Marbach I, Mayer AM. 1981. Properties of gallic acid-induced extracellular laccase of *Botrytis cinerea*. Phytochemistry. 20:1211–1213.

[cit0016] Gomaa OM, Momtaz OA. 2015. Copper induction and differential expression of laccase in *Aspergillus flavus*. Braz J Microbiol. 46:285–292. 26221119 10.1590/S1517-838246120120118 PMC4512072

[cit0017] Guetsky R, Kobiler I, Wang X, Perlman N, Gollop N, Avila-Quezada G, Hadar I, Prusky D. 2005. Metabolism of the flavonoid epicatechin by laccase of *Colletotrichum gloeosporioides* and its effect on pathogenicity on avocado fruits. Phytopathology. 95:1341–1348. 18943366 10.1094/PHYTO-95-1341

[cit0018] Guillén Y, Machuca Á. 2008. The effect of copper on the growth of wood-rotting fungi and a blue-stain fungus. World J Microbiol Biotechnol. 24:31–37.

[cit0019] Jang MY, Ryu WY, Cho MH. 2006. Enhanced production of laccase from*Trametes sp*. by combination of various inducers. Biotechnol Bioprocess Eng. 11:96–99.

[cit0020] Jin W, Wu F. 2015. Characterization of miRNAs associated with *Botrytis cinerea* infection of tomato leaves. BMC Plant Biol. 15:1. 25592487 10.1186/s12870-014-0410-4 PMC4311480

[cit0021] Kannaiyan R, Mahinpey N, Mani T, Martinuzzi RJ, Kostenko V. 2012. Enhancement of *Dichomitus squalens* tolerance to copper and copper-associated laccase activity by carbon and nitrogen sources. Biochem Eng J. 67:140–147.

[cit0022] Kelloniemi J, Trouvelot S, Heloir MC, Simon A, Dalmais B, Frettinger P, Cimerman A, Fermaud M, Roudet J, Baulande S, et al. 2015. Analysis of the molecular dialogue between gray mold (*Botrytis cinerea*) and grapevine (*Vitis vinifera*) reveals a clear shift in defense mechanisms during berry ripening. Mol Plant-Microbe Interact. 28:1167–1180. 26267356 10.1094/MPMI-02-15-0039-R

[cit0023] Kim YH, Lee HS, Kwon HJ, Patnaik BB, Nam KW, Han YS, Bang IS, Han MD. 2014. Effects of different selenium levels on growth and regulation of laccase and versatile peroxidase in white-rot fungus, *Pleurotus eryngii*. World J Microbiol Biotechnol. 30:2101–2109. 24643367 10.1007/s11274-014-1636-x

[cit0024] Kuhar F, Papinutti L. 2014. Optimization of laccase production by two strains of *Ganoderma lucidum* using phenolic and metallic inducers. Rev Argent Microbiol. 46:144–149. 25011599 10.1016/S0325-7541(14)70063-X

[cit0025] Laemmli UK. 1970. Cleavage of structural proteins during the assembly of the head of bacteriophage T4. Nature. 227:680–685. 5432063 10.1038/227680a0

[cit0026] Litvintseva AP, Henson JM. 2002. Cloning, characterization, and transcription of three laccase genes from *Gaeumannomyces graminis* var. *tritici*, the take-all fungus. Appl Environ Microbiol. 68:1305–1311. 11872481 10.1128/AEM.68.3.1305-1311.2002 PMC123725

[cit0027] Livak KJ, Schmittgen TD. 2001. Analysis of relative gene expression data using real-time quantitative PCR and the 2(-Delta Delta C(T)) method. Methods. 25:402–408. 11846609 10.1006/meth.2001.1262

[cit0028] Lorenzini M, Azzolini M, Tosi E, Zapparoli G. 2013. Postharvest grape infection of *Botrytis cinere*a and its interactions with other moulds under withering conditions to produce noble-rotten grapes. J Appl Microbiol. 114:762–770. 23163324 10.1111/jam.12075

[cit0029] Maestre-Reyna M, Liu WC, Jeng WY, Lee CC, Hsu CA, Wen TN, Wang AHJ, Shyur L. 2015. Structural and functional roles of glycosylation in fungal laccase from *Lentinus* sp. PLoS One. 10(4):e0120601. 25849464 10.1371/journal.pone.0120601 PMC4388643

[cit0030] Makela MR, Lundell T, Hatakka A, Hildén K. 2013. Effect of copper, nutrient nitrogen, and wood-supplement on the production of lignin-modifying enzymes by the white-rot fungus phlebia radiata. Fungal Biol. 117:62–70. 23332834 10.1016/j.funbio.2012.11.006

[cit0031] Manavalan T, Manavalan A, Thangavelu KP, Heese K. 2013. Characterization of optimized production, purification and application of laccase from *Ganoderma lucidum*. Biochem Eng J. 70:106–114.

[cit0032] Manzl C, Enrich J, Ebner H, Dallinger R, Krumschnabel G. 2004. Copper-induced formation of reactive oxygen species causes cell death and disruption of calcium homeostasis in trout hepatocytes. Toxicology. 196:57–64. 15036756 10.1016/j.tox.2003.11.001

[cit0033] Marbach I, Harel E, Mayer AM. 1984. Molecular properties of extracellular *Botrytis cinerea* laccase. Phytochemistry. 23:2713–2717.

[cit0034] Morozova OV, Shumakovich GP, Gorbacheva MA, Shleev SV, Yaropolov AI. 2007. “Blue” laccases. Biochemistry (Mosc). 72:1136–1150. 18021071 10.1134/s0006297907100112

[cit0035] Nakajima M, Akutsu K. 2014. Virulence factors of *Botrytis cinerea*. J Gen Plant Pathol. 80:15–23.

[cit0036] Palmieri G, Giardina P, Bianco C, Fontanella B, Sannia G. 2000. Copper induction of laccase isoenzymes in the ligninolytic fungus *Pleurotus ostreatus*. Appl Environ Microbiol. 66:920–924. 10698752 10.1128/aem.66.3.920-924.2000 PMC91923

[cit0037] Park JW, Kang HW, Ha BS, Kim SI, Kim S, Ro HS. 2015. Strain-dependent response to Cu^2+^ in the expression of laccase in *Pycnoporus coccineus*. Arch Microbiol. 197:589–596. 25677944 10.1007/s00203-015-1090-7

[cit0038] Passarini MR, Ottoni CA, Santos C, Lima N, Sette LD. 2015. Induction, expression and characterisation of laccase genes from the marine-derived fungal strains *Nigrospora* sp. CBMAI 1328 and *Arthopyrenia* sp. CBMAI 1330. AMB Express. 5:19. 25852996 10.1186/s13568-015-0106-7 PMC4385153

[cit0039] Piscitelli A, Giardina P, Lettera V, Pezzella C, Sannia G, Faraco V. 2011. Induction and transcriptional regulation of laccases in fungi. Curr Genomics. 12:104–112. 21966248 10.2174/138920211795564331 PMC3129044

[cit0040] Ramirez-Cavazos LI, Junghanns C, Nair R, Cardenas-Chavez DL, Hernandez-Luna C, Agathos SN, Parra R. 2014. Enhanced production of thermostable laccases from a native strain of *Pycnoporus sanguineus* using central composite design. J Zhejiang Univ Sci B. 15:343–352. 24711355 10.1631/jzus.B1300246 PMC3989153

[cit0041] Rayner ADM, Boddy L. 1988. Fungal decomposition of wood: its biology and ecology. Chichester, Sussex, UK: John Wiley & Sons Ltd.

[cit0042] Rha E, Park HJ, Kim MO, Chung YR, Lee CW, Kim JW. 2001. Expression of exo-polygalacturonases in *Botrytis cinerea*. FEMS Microbiol Lett. 201:105–109. 11445175 10.1111/j.1574-6968.2001.tb10740.x

[cit0043] Sbaghi M, Jeandet P, Bessis R, Leroux P. 1996. Degradation of stilbene-type phytoalexins in relation to the pathogenicity of *Botrytis cinerea* to grapevines. Plant Pathol. 45:139–144.

[cit0044] Schouten A, Wagemakers L, Stefanato FL, van der Kaaij RM, van Kan JAL. 2002. Resveratrol acts as a natural profungicide and induces self-intoxication by a specific laccase. Mol Microbiol. 43:883–894. 11929539 10.1046/j.1365-2958.2002.02801.x

[cit0045] Shah P, Gutierrez-Sanchez G, Orlando R, Bergmann C. 2009. A proteomic study of pectin-degrading enzymes secreted by *Botrytis cinerea* grown in liquid culture. Proteomics. 9:3126–3135. 19526562 10.1002/pmic.200800933 PMC2761233

[cit0046] Simon A, Dalmais B, Morgant G, Viaud M. 2013. Screening of a *Botrytis cinerea* one-hybrid library reveals a Cys2His2 transcription factor involved in the regulation of secondary metabolism gene clusters. Fungal Genet Biol. 52:9–19. 23396263 10.1016/j.fgb.2013.01.006

[cit0047] Slomczynski D, Nakas JP, Tanenbaum SW. 1995. Production and characterization of laccase from *Botrytis cinerea* 61-34. Appl Environ Microbiol. 61:907–912. 16534974 10.1128/aem.61.3.907-912.1995 PMC1388373

[cit0048] Solomon EI, Sundaram UM, Machonkin TE. 1996. Multicopper oxidases and oxygenases. Chem Rev. 96:2563–2606. 11848837 10.1021/cr950046o

[cit0049] Stohs SJ, Bagchi D. 1995. Oxidative mechanisms in the toxicity of metal ions. Free Radic Biol Med. 18:321–336. 7744317 10.1016/0891-5849(94)00159-h

[cit0050] Thurston CF. 1994. The structure and function of fungal laccases. Microbiology-Sgm. 140:19–26.

[cit0051] Valette-Collet O, Cimerman A, Reignault P, Levis C, Boccara M. 2003. Disruption of *Botrytis cinerea* pectin methylesterase gene Bcpme1 reduces virulence on several host plants. Mol Plant Microbe Interact. 16:360–367. 12744465 10.1094/MPMI.2003.16.4.360

[cit0052] Van Kan JA, Stassen JH, Mosbach A, Van Der Lee TA, Faino L, Farmer AD, Papasotiriou DG, Zhou S, Seidl MF, Cottam E., et al. 2017. A gapless genome sequence of the fungus botrytis cinerea. Mol Plant Pathol. 18:75–89. 26913498 10.1111/mpp.12384 PMC6638203

[cit0053] Vincent D, Wheatley MD, Cramer GR. 2006. Optimization of protein extraction and solubilization for mature grape berry clusters. Electrophoresis. 27:1853–1865. 16586412 10.1002/elps.200500698

[cit0054] Viswanath B, Chandra MS, Kumar KP, Pallavi H, Reddy BR. 2008. Fungal laccases and their biotechnological applications with special reference to bioremediation. Dyn Biochem Process Biotech Mol Biol. 2:1–13.

[cit0055] Viswanath B, Rajesh B, Janardhan A, Kumar AP, Narasimha G. 2014. Fungal laccases and their applications in bioremediation. Enzyme Res. 2014:21. 10.1155/2014/163242 PMC4052089 24959348

[cit0056] Williamson B, Tudzynski B, Tudzynski P, van Kan JA. 2007. *Botrytis cinerea*: the cause of grey mould disease. Mol Plant Pathol. 8:561–580. 20507522 10.1111/j.1364-3703.2007.00417.x

[cit0057] Yaropolov AI, Skorobogat’ko OV, Vartanov SS, Varfolomeyev SD. 1994. Laccase. Appl Biochem Biotechnol. 49:257–280.

