# Supplementary material for: Copper induces transcription of BcLCC2 laccase gene in phytopathogenic fungus, Botrytis cinerea
Source: Mycology. 2020 Feb 11;12(1):48–57. doi: 10.1080/21501203.2020.1725677 (PMC7889114; doi:10.1080/21501203.2020.1725677)
Supplement: Supplemental Material [file TMYC_A_1725677_SM7185.docx]

**Supplementary method**

**NanoLC ESI MS/MS data acquisition**

**Instruments used**

Mass spectrometer: Triple TOF 5600 (AB Sciex) NanoLC system: Eksigent Ultra nanoLC system (Eksigent) Analytical column: Halo C18, 160Å, 2.7μm, 150 μm x 10 cm

**Sample preparation:** The largest of the gel bands were cut and destained with acetonitrile/ammonium bicarbonate at 37^o^C. Gels were reduced with 25 mM DTT at 56^o^C for 30 min. Proteins were alkylated with 55 mM iodoacetamide for 20 min in dark. Each sample was digested for 16 h with 250 ng of trypsin. Gels were sonicated, washed with acetonitrile/formic acid and followed by drying these extractions. Samples were reconstituted into 20 μL loading buffer.

**NanoLC ESI MS/MS data acquisition:** Sample (10 μL) was injected onto a peptide trap (NanoLCMS peptide cap trap) for pre-concentration and desalted with 0.1% formic acid, 2% ACN, at 5 μL/min for 4 minutes. The peptide trap was then switched into line with the analytical column. Peptides were eluted from the column using a linear solvent gradient, with steps, from H2O:CH3CN (98:2; + 0.1% formic acid) to H2O:CH3CN (2:98; + 0.1% formic acid) with constant flow (600 nL/min) over an 80 min period. The LC eluent was subject to positive ion Nano flow electrospray MS analysis in an information dependent acquisition mode (IDA).

**Commercial in confidence:** In the IDA mode a TOFMS survey scan was acquired (m/z 350-1500, 0.25 seconds), with ten largest multiply charged ions (counts >150) in the survey scan sequentially subjected to MS/MS analysis. MS/MS spectra were accumulated for 100 milli-seconds (m/z 100-1500) with rolling collision energy.

**Data processing:** The raw data files (.wiff) were converted to mascot generic files (.mgf)

using AB SCIEX CommandDriver software. They were submitted to Mascot

(Matrix Science, UK) and searched against SwissProt Fungi database

(32,471 sequences).

Database : SwissProt 2016_10 (552884 sequences; 197760918 residues)

Taxonomy : Fungi (32471 sequences)

Type of search : MS/MS Ion Search

Enzyme : Trypsin

Variable modifications : Carbamidomethyl (C),Oxidation (M)

Mass values : Monoisotopic

Protein Mass : Unrestricted

Peptide Mass Tolerance : ± 50 ppm

Fragment Mass Tolerance: ± 0.1 Da

Max Missed Cleavages : 1

Instrument type : ESI-QUAD-TOF

**Supplementary table:** RT-qPCR primers for *Botrytis cinerea* laccases (*BcLCC1*, *BcLCC2*, *BcLCC3*) and the housekeeping gene (ActinA) used in the study.

| **Target** | **Forward (5’-3’)** | **Reverse (5’-3’)** |
| --- | --- | --- |
| *B. cinerea* actin (ActinA) | GGTCTTGAGAGCGGTGGTAT | CGTCCTGTAAACTTCGCAGA |
| *B. cinerea* laccase 1 (*BcLCC1*) | TCAGTACGACGGAGTTCCAG | ACCATAGTTGTCCGCATGAA |
| *B. cinerea* laccase 2 (*BcLCC*2) | TCCAAGATTGGGCACATAAA | ACCAACGCAGTTAGCATCAG |
| *B. cinerea* laccase 3 (*BcLCC*3) | CCCATTGTTCCATACACTGC | TTTGAACTTCAGCACGGAAC |
